# Euploid miscarriage is associated with elevated serum C-reactive protein levels in infertile women: a pilot study

**DOI:** 10.1007/s00404-020-05461-1

**Published:** 2020-02-27

**Authors:** Andrea Weghofer, David H. Barad, Sarah K. Darmon, Vitaly A. Kushnir, David F. Albertini, Norbert Gleicher

**Affiliations:** 1 Department of Obstetrics and Gynecology, Medical University of Vienna, Vienna, Austria; 2 The Center for Human Reproduction, New York, N.Y. USA; 3 The Foundation for Reproductive Medicine, New York, N.Y. USA; 4 Department of Obstetrics and Gynecology, Wake Forest University, Winston Salem, N.C. USA; 5 Stem Cell Biology and Molecular Embryology Laboratory, The Rockefeller University, New York, N.Y USA

**Keywords:** C-reactive protein (CRP), Euploid, Infertility, Inflammation, Miscarriage

## Abstract

**Purpose:**

Increased serum C-protein (CRP) levels reduce fecundity in healthy eumenorrheic women with 1–2 pregnancy losses. Subclinical systemic inflammation may impede maternal immune tolerance toward the fetal semi-allograft, compromising implantation and early embryonic development. Some miscarriages with normal karyotypes could, therefore, be caused by inflammation. Whether pre-pregnancy CRP relates to karyotypes of spontaneously aborted products of conception (POCs) was investigated.

**Methods:**

A study cohort of 100 infertile women with missed abortions who underwent vacuum aspirations followed by cytogenetic analysis of their products of conception tissue was evaluated at an academically affiliated fertility center. Since a normal female fetus cannot be differentiated from maternal cell contamination (MCC) in conventional chromosomal analyses, POC testing was performed by chromosomal microarray analysis. MCC cases and incomplete data were excluded. Associations of elevated CRP with first trimester pregnancy loss in the presence of a normal fetal karyotype were investigated.

**Results:**

Mean patients’ age was 39.9 ± 5.8 years; they demonstrated a BMI of 23.9 ± 4.6 kg/m^2^ and antiMullerian hormone (AMH) of 1.7 ± 2.4 ng/mL; 21.3% were parous, 19.1% reported no prior pregnancy losses, 36.2% 1–2 and 6.4% ≥ 3 losses. Karyotypes were normal in 34% and abnormal in 66%. Adjusted for BMI, women with elevated CRP were more likely to experience euploid pregnancy loss (*p* = 0.03). This relationship persisted when controlled for female age and AMH.

**Conclusions:**

Women with elevated CRP levels were more likely to experience first trimester miscarriage with normal fetal karyotype. This relationship suggests an association between subclinical inflammation and miscarriage.

## Introduction

The detrimental effects of chronic subclinical inflammation on human health are increasingly becoming apparent. Mediating vascular remodeling, inflammation was recently recognized to induce and promote cardiovascular disease [1]. Biomarkers of micro-inflammation, such as C-reactive protein (CRP), serve as predictive tools for the development and monitoring of the acute coronary syndrome, as inflammation is considered a target that can be therapeutically improved [3, 13]. These insights have improved the understanding of how inflammation promotes a variety of pregnancy-induced disorders, such as gestational diabetes and pre-eclampsia [23, 29]. Anti-inflammatory drugs, such as aspirin, have proven beneficial in preventing and mitigating pre-eclampsia-associated adverse pregnancy outcomes in expectant mothers at risk [17].

Preconception and inflammation play an important physiological role in ovulation and implantation [14, 24] but, increasingly, thus also appear to exert potentially detrimental effects on reproductive success. To allow for implantation and proper development of the embryonic semi-allograft, the female immune system must induce tolerance. In the presence of micro-inflammation, induction of fetal–maternal tolerance may be derailed, increasing risks of subfertility, repeated implantation failure and pregnancy loss [6, 10, 12].

Radin et al. [20] recently reported impaired fecundity in eumenorrheic women with increased CRP levels. Their findings are supported by the reported effects of aspirin in the Gestation and Reproduction (EAGER) trial which demonstrated that low-dose aspirin restored pregnancy and live birth rates among eumenorrheic women with one or two pregnancy losses and abnormally high preconception CRP [25]. Subclinical inflammations could, thus, serve as a causative factor for the substantial proportion of miscarriages that reveal normal karyotypes after the analysis of products of conception (POCs) [8]. If this were, indeed, the case, normal POC karyotypes should be more prevalent in women with abnormally high CRP levels.

Conventional chromosomal analyses of POC-karyotypes do not allow discrimination between normal female karyotypes and maternal cell contaminations (MCCs). The introduction of chromosomal microarray analysis, featuring single nucleotide polymorphism technology (Anora®, Natera, San Carlos, CA), however, now facilitates correct differentiation.

This study was initiated to shed further light on the relationship between maternal micro-inflammation, reflected in pre-pregnancy CRP levels, and POC karyotypes.

## Methods

We retrospectively evaluated 100 consecutive infertile women who presented with missed abortions and underwent dilatation and curettage (D and C) with cytogenetic analysis of POCs at The Center for Human Reproduction (CHR) in New York City, NY. Patients with abnormal parental karyotypes, genetic disorders, uterine malformations, autoimmune disorders, such as antiphospholipid syndrome, chronic endometritis or clinical signs of spontaneous abortion were not eligible for enrollment.

In the course of routine workup for fertility treatment, women underwent vaginal sonography, baseline hormone testing of follicle stimulating (FSH) and antiMullerian hormone (AMH), estradiol and androgen serum levels on cycle days 2/3. As part of their routine workup for fertility treatment, patients at our center also undergo CRP assessments. Fertility treatment was performed as previously reported [11]. Five to six weeks after the first positive serum human chorionic gonadotropin (hCG), vaginal ultrasound was performed. Missed abortions were diagnosed by the presence of at least one gestational sac and absence of fetal heart activity. Before surgical evacuation, all women signed case-specific informed consents for surgery and chromosomal analyses of POCs. Evacuation was performed by suction aspiration within days from diagnosis. Patients’ blood and POCs were collected under sterile conditions, processed and shipped to a national reference laboratory as indicated in the test provider’s instructions of the sample kit (Anora®, Natera, San Carlos, CA). Testing was performed by microarray analysis, featuring single nucleotide polymorphism technology (Anora®, Natera, San Carlos, CA).

**Fig. 1:**
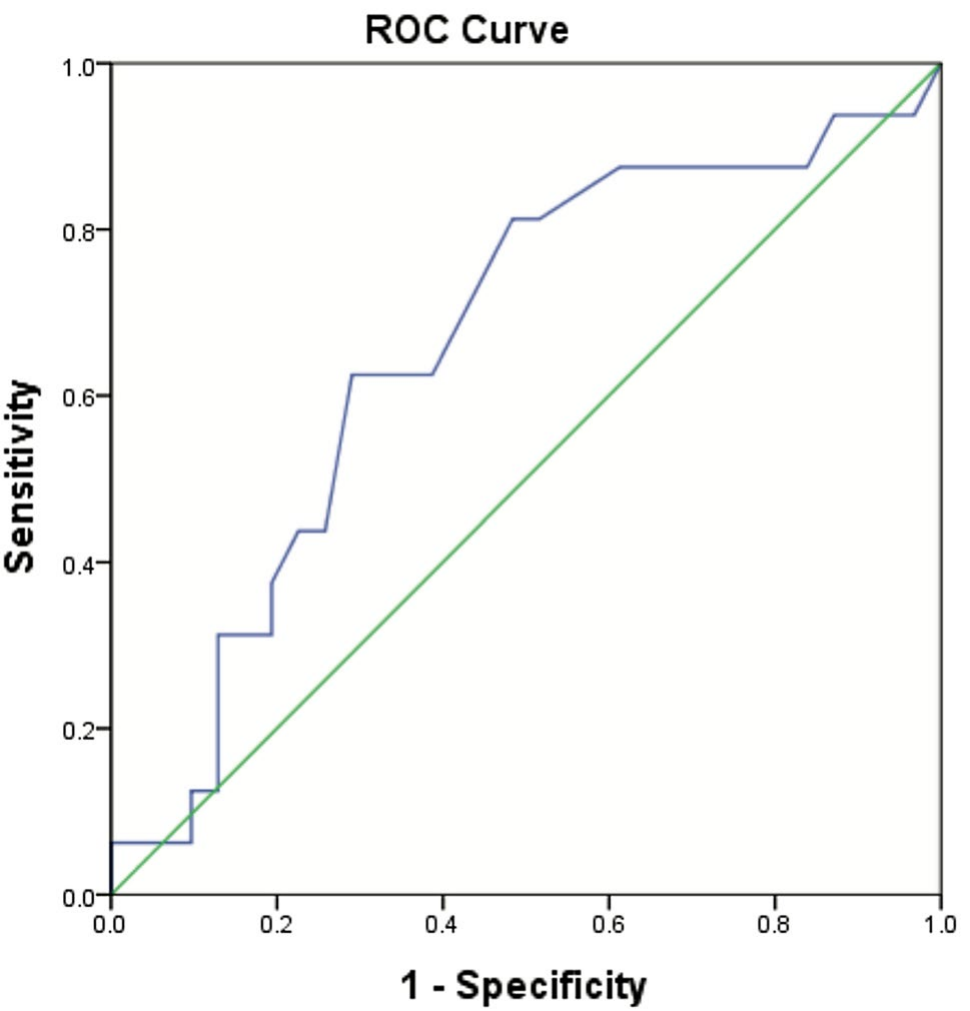
CRP and fetal karyotype. ROC curve demonstrating the relationship between CRP and fetal karyotype

Serum CRP levels increase with age and body mass index (BMI) [15]. The EAGER trial assessed preconception CRP levels in healthy women attempting pregnancy. In this large US trial, women without low-grade inflammation (i.e., low CRP tertile) were defined as CRP < 0.73 mg/L [25]. In this study, area under the curve (AUC) for CRP of aneuploid pregnancy loss was 0.66 ± 0.09 (*p* = 0.07) (Fig. 1). Based on this ROC curve, the maximal inflection point (81% sensitivity, 51.6% specificity) was around CRP 0.75 mg/L and, therefore, practically identical to the EAGER trial [13]. In this study, patients with CRP of 0.75 mg/L were, therefore, considered to have an elevated CRP. Study subjects with incomplete data sets (i.e., *n =* 35) and/or POC analyses that revealed MCC (i.e., *n =* 16) or inconclusive results (i.e., *n =* 2) were excluded from further analysis. This resulted in 47 women with complete data for final analysis.

Continuous data were expressed as mean ± SD and compared via ANOVA. Outcome parameters are presented as proportions. The association of elevated CRP with euploid pregnancy loss was tested using logistic regression, adjusting for appropriate covariates. All statistical analyses were carried out with use of the Statistical Package for the Social Sciences 21.0 (SPSS). A *p* < 0.05 was considered statistically significant.

At initial consultation, patients at our center sign an informed consent, which allows for the use of their anonymized electronic medical records and, where needed, of their paper records, if the patient’s identity remains protected and the medical record remains confidential. Since here-presented data only involved retrospective review of the center’s anonymized electronic research database, also used to report the center’s annual IVF outcomes to national registries, these conditions were met. The Center for Human Reproduction Institutional Review Board approved such medical record studies.

## Results

The women studied were 39.9 ± 5.8 years old. They presented with a BMI of 23.5 ± 4.7 kg/m^2^, AMH of 1.7 ± 2.4 ng/mL and CRP of 2.0 ± 2.7 mg/L. There was no significant difference in age, BMI, AMH levels, semen analysis parameters or white blood cell counts between couples with euploid and aneuploid pregnancy loss (Table 1). All women were taking prednisone during pregnancy and, thus, WBC counts while pregnant were significantly higher than when not pregnant. Within the study cohort, 29/47 (61.7%) had previous pregnancies, 10/47 (21.3%) had a previous live birth, 9/47 (19.1%) had no prior pregnancy losses, 17/47 (36.2%) had 1–2 prior first trimester miscarriages and 3/47 (6.4%) had a history of three or more losses. Thirty-three women presented with normal BMI, ten were overweight and four suffered from obesity.

**Table 1:** Patient characteristics according to fetal karyotype in 47 women with first trimester miscarriage

	All	Euploid	Aneuploid
	(*n =* 47)	(*n =* 16)	(*n =* 31)
Female age (years)	39.9 ± 5.8	39.1 ± 7.3	40.3 ± 4.9
BMI (kg/m^2^)	23.6 ± 4.6	23.7 ± 4.3	23.6 ± 4.9
AMH (ng/mL)	1.7 ± 2.4	2.1 ± 3.3	1.5 ± 1.8
CRP (mg/L)	2.2 ± 3.0	2.9 ± 3.3	1.9 ± 2.7
Semen analysis			
Vol	1.9 ± 1.3	1.9 ± 1.7	2.0 ± 1.3
Count (× 10^6^)	58.0 ± 63.1	50.3 ± 46.4	62.0 ± 72.0
Motility (%)	43.0 ± 21.0	46.0 ± 17.4	62.0 ± 72.0
Morphology(%)	11 ± 19	8 ± 5	12 ± 24
WBC			
Before pregnancy	4.4 ± 6.3	4.7 ± 7	3.8 ± 6.4
During SAB	8.8 ± 11.5	7.3 ± 11.8	8.7 ± 12.3

Values are presented as means ± SD

Indications for fertility treatment were diminished ovarian reserve in 40 women, male factor infertility in 10 and tubal factor in 4 couples, 2 patients suffered from polycystic ovary syndrome (PCOS) and one had endometriosis (some patients had multiple diagnoses). None of the study subjects suffered from hypertension or diabetes. Within the study cohort, 43 women conceived via assisted reproduction (i.e., IVF or ICSI), 2 in the course of controlled ovarian hyperstimulation and intrauterine insemination (IUI) and the remaining 2 conceived spontaneously during work up for fertility treatment. Sixteen patients (34%) showed a normal fetal karyotype in their POC analysis, while 31 patients (66%) presented with aneuploid pregnancy losses. Details on karyotypes of women with aneuploid conceptions are presented in Table 2.

**Table 2:** Karyotypes of product of conception analyses in women undergoing miscarriage

Karyotype	Count	%
46,XY	9	14.29
46,XX	7	11.11
46,XX UPD 1–22,X	1	1.59
45,X0	1	1.59
47,XX + 7	1	1.59
47,XX + 8	1	1.59
47,XX + 11	1	1.59
47,XX + 15	2	3.17
47,XX + 16	2	3.17
47,XX + 22	1	1.59
47,XY + 3	3	4.76
47,XY + 9	1	1.59
47,XY + 15	1	1.59
47,XY + 16	2	3.17
47,XY + 16 mosaic	1	1.59
47,XY + 22	1	1.59
48,XX + 4, + 16	1	1.59
48,XY + 10, + 18	1	1.59
48,XY + 14, + 22	1	1.59
48,XY + 21, + 22	1	1.59
49,XX + 9, + 18, + 20	1	1.59
69,XX + 2	1	1.59
69,XXX maternal	3	4.76
69,XXY maternal	2	3.17
72,XXY + 5, + 6, + 21	1	1.59
MCC	16	25.4

Age, BMI and AMH have all been previously reported to be associated with changes in CRP levels [18]. To evaluate a possible association between CRP, age, BMI and AMH, regression analyses were performed. Only BMI demonstrated a significant positive relationship with CRP levels (*p* < 0.01) and was, therefore, included in the multi-regression model. After adjustment for BMI, POCs of women with elevated CRP levels were significantly more likely to demonstrate normal fetal karyotypes (*p* = 0.03). Odds of having a euploid embryo loss were 5.5 (95 CI 1.2–25.2) in women with elevated CRP levels and this association remained significant when further adjusted for female age and AMH levels.

## Discussion

In this study, patients with elevated preconception CRP concentrations were more likely to demonstrate normal karyotypes than women with normal CRP levels. These findings suggest that subclinical inflammation may create challenges for the maternal immune system to reprogram itself towards tolerance. Similar conclusions were also reached by Barad et al. [2], when discussing a recent miscarriage study by Feichtinger et al. [9] who reported patients with recurrent miscarriage demonstrated less aneuploidy than those with sporadic miscarriage. Repeat aborters, therefore, appeared to have more non-chromosomal causes for pregnancy loss, likely mostly immune-mediated losses.

Most recent studies suggest that aneuploidy maximally represent about 50% of causes of fetal demise [26]. A considerable number of pregnancies with normal karyotypes, thus, result in miscarriages. In this study, practically two-third of miscarriages were aneuploid, though this number is, likely, consequence of above average age of our center’s patient population. While associations between miscarriages and specific conditions, such as antiphospholipid syndrome or uterine malformations, are well established [19, 28], underlying mechanisms for most other non-chromosomal miscarriages are still only poorly understood.

Increased miscarriage rates in women with overt autoimmune conditions offer support for inflammation potentially impeding the crosstalk between fetus and maternal immune system at the maternal–fetal interface [5, 21]. If even subclinical inflammation, reflected in elevated CRP levels in absence of systemic inflammatory symptoms, impairs fetal growth and development, any excessive degree of inflammatory response may hinder timely induction of tolerance.

A number of observations suggest that the timeline for physiological tolerance pathways starts prior to conception: before human embryos implant, they spend about 2 days within the uterine cavity. This time frame, therefore, likely defines a period of crucially important crosstalk between embryo, endometrium and the maternal immune system. Embryos, however, also are able to implant extrauterine and even in vitro [7]. It, thus, seems likely that the initial signal that creates a welcoming implantation environment may come from the embryo.

Endometrium (or other sites in cases of extrauterine pregnancies), in turn, must react properly by abandoning its absolute hostility toward the invasion of any kind (an absolutely essential quality of the endometrium that routinely must protect women from trans-endometrial bacterial, viral and parasitic infections), and, instead, creates a welcoming microenvironment for invasiveness (i.e., implantation). Since implantation sites never demonstrate allogeneic immune responses, the process must include development of an initial level of tolerance by the maternal immune system that prevents immediate rejection of the invading embryo. This concept of required timely induction of tolerance pathways is supported by improved fecundity in chronically infected females with distinct helminthic infections, which likely induce common tolerance pathways to implanting embryos [4].

If induction of pregnancy tolerance, indeed, follows a prefixed timeline, one can also assume that some miscarriages reflect insufficient tolerance induction, with the consequence of allogeneic immune responses of the maternal immune system against the implanting embryo. As hyperactive immune systems are known to hamper induction of tolerance pathways, increased miscarriage risk (and, possibly, implantation failure) with laboratory evidence of inflammation should not surprise. All inflammatory markers may, however, not denote identical risks, just as all helminths do not induce the same tolerance pathways [4].

If subclinical inflammation can lead to insufficient induction of tolerance, anti-inflammatory drugs should increase reproductive health by improving pregnancy rates, reducing miscarriage and, maybe, even ameliorating pre-eclampsia in women at risk. Large double-blind placebo-controlled trials support this hypothesis. Sjaarda et al. recently reported increased implantation and live birth chances after low-dose aspirin treatment among eumenorrheic women with a history of one or two pregnancy losses and increased preconception CRP levels. In contrast, such an impact was absent with normal preconception CRP [25]. Similar results are also reported after low-dose prenatal aspirin treatment in women at risk for pre-eclampsia. Rolnik et al. [22] described a 62% risk reduction for preterm preeclampsia after low-dose aspirin treatment in at risk populations.

That timing of when anti-inflammatory treatment should be started is crucial for its success, further supports the timeline concept of tolerance induction: Tempfer et al. [27] reported higher live birth rates in women with idiopathic recurrent miscarriage after prednisone, low-dose aspirin and progesterone treatment initiated preconceptionally. Moore et al. [16] demonstrate similar effects with pre-eclampsia: low-dose aspirin significantly reduced pre-eclampsia risk when treatment was initiated before week 17, long before initial symptoms of pre-eclampsia became apparent. That aspirin exerts benefits where vascular remodeling and chronic inflammation interphase, emphasizes their close interplay and its importance for implantation and the exponential growth of products of conception during gestation.

Here-presented evidence, thus, further suggests that preconception assessments of CRP, a widely available and low-cost blood test, may in infertile women be useful in identifying those at increased risk for immune system-induced miscarriages which, in turn, may allow for timely pharmaceutical interventions. Prospectively randomized studies are, however, required to confirm this hypothesis.

The retrospective nature of this study is an obvious weakness. Though reflecting an unselected patient population, retrospective studies can never be absolutely free of selection biases. This weakness is, however, compensated by the fact that patients with and without abnormally high CRP levels were similar in important patient characteristics.

This study raises two immediate challenges: first, the question arises whether other inflammatory markers are equally or, maybe, even more predictive of treatment outcomes in infertility. Second, prospective randomized studies are necessary to determine in women with abnormally high CRP (and potentially other inflammatory markers) which anti-inflammatory medications will be most effective in counteracting adverse effects on female reproduction. In conclusion, women with elevated CRP levels were more likely to experience first trimester miscarriage with normal fetal karyotype. This relationship suggests an association between subclinical inflammation and miscarriage.
